# Upregulation of GALNT7 in prostate cancer modifies *O*-glycosylation and promotes tumour growth

**DOI:** 10.1038/s41388-023-02604-x

**Published:** 2023-02-01

**Authors:** Emma Scott, Kirsty Hodgson, Beatriz Calle, Helen Turner, Kathleen Cheung, Abel Bermudez, Fernando Jose Garcia Marques, Hayley Pye, Edward Christopher Yo, Khirul Islam, Htoo Zarni Oo, Urszula L. McClurg, Laura Wilson, Huw Thomas, Fiona M. Frame, Margarita Orozco-Moreno, Kayla Bastian, Hector M. Arredondo, Chloe Roustan, Melissa Anne Gray, Lois Kelly, Aaron Tolson, Ellie Mellor, Gerald Hysenaj, Emily Archer Goode, Rebecca Garnham, Adam Duxfield, Susan Heavey, Urszula Stopka-Farooqui, Aiman Haider, Alex Freeman, Saurabh Singh, Edward W. Johnston, Shonit Punwani, Bridget Knight, Paul McCullagh, John McGrath, Malcolm Crundwell, Lorna Harries, Denisa Bogdan, Daniel Westaby, Gemma Fowler, Penny Flohr, Wei Yuan, Adam Sharp, Johann de Bono, Norman J. Maitland, Simon Wisnovsky, Carolyn R. Bertozzi, Rakesh Heer, Ramon Hurtado Guerrero, Mads Daugaard, Janne Leivo, Hayley Whitaker, Sharon Pitteri, Ning Wang, David J. Elliott, Benjamin Schumann, Jennifer Munkley

**Affiliations:** 1 Newcastle University Centre for Cancer, Newcastle University Institute of Biosciences, Newcastle, NE1 3BZ UK; 2 The Chemical Glycobiology Laboratory, The Francis Crick Institute, NW1 1AT London, UK; 3 Department of Chemistry, Imperial College London, W12 0BZ London, UK; 4 Cellular Pathology, The Royal Victoria Infirmary, Queen Victoria Road, Newcastle upon Tyne, NE1 4LP UK; 5 Canary Center at Stanford for Cancer Early Detection, Department of Radiology, Stanford University, Palo Alto, CA 94304 USA; 6 Molecular Diagnostics and Therapeutics Group, Charles Bell House, Division of Surgery and Interventional Science, University College London, London, UK; 7 Department of Life Technologies, Division of Biotechnology, University of Turku, Turku, Finland; 8 Department of Urologic Sciences, University of British Columbia, Vancouver, BC V5Z 1M9 Canada; 9 Vancouver Prostate Centre, Vancouver, BC V6H 3Z6 Canada; 10 Institute for Integrative Biology, University of Liverpool, Liverpool, L69 7ZB UK; 11 Newcastle University Centre for Cancer, Translational and Clinical Research Institute, Paul O’Gorman Building, Newcastle University, Newcastle upon Tyne, NE2 4HH UK; 12 Cancer Research Unit, Department of Biology, University of York, Heslington, North Yorkshire, YO10 5DD UK; 13 The Mellanby Centre for Musculoskeletal Research, Department of Oncology and Metabolism, The University of Sheffield, Sheffield, UK; 14 Structural Biology Science Technology Platform, The Francis Crick Institute, NW1 1AT London, UK; 15 Sarafan Chem-H and Departemnt of Chemistry, Stanford University, 424 Santa Teresa St, Stanford, CA 94305 USA; 16 Department of Pathology, UCLH NHS Foundation Trust, London, UK; 17 UCL Centre for Medical Imaging, Charles Bell House, University College London, London, UK; 18 NIHR Exeter Clinical Research Facility, Royal Devon and Exeter NHS Foundation Trust, Exeter, UK; 19 Department of Pathology, Royal Devon and Exeter NHS Foundation Trust, Exeter, UK; 20 Exeter Surgical Health Services Research Unit, Royal Devon and Exeter NHS Foundation Trust, Exeter, UK; 21 Institute of Biomedical and Clinical Sciences, Medical School, College of Medicine and Health, University of Exeter, Exeter, UK; 22 Division of Clinical Studies, The Institute of Cancer Research, London, SM2 5NG UK; 23 Prostate Cancer Targeted Therapy Group, The Royal Marsden Hospital, London, SM2 5PT UK; 24 University of British Columbia, Faculty of Pharmaceutical Sciences, Vancouver, BC V6T 1Z3 Canada; 25 Howard Hughes Medical Institute, 424 Santa Teresa St, Stanford, CA 94305 USA; 26 Department of Urology, Freeman Hospital, The Newcastle upon Tyne Hospitals NHS Foundation Trust, Newcastle upon Tyne, NE7 7DN UK; 27 University of Zaragoza, Mariano Esquillor s/n, Campus Rio Ebro, Edificio I+D, Zaragoza, Spain; Fundación ARAID, 50018 Zaragoza, Spain; 28 Copenhagen Center for Glycomics, Department of Cellular and Molecular Medicine, University of Copenhagen, Copenhagen, Denmark; 29 InFLAMES Research Flagship Center, University of Turku, Turku, Finland

**Keywords:** Prostate cancer, Glycobiology, Diagnostic markers, Cell growth

## Abstract

Prostate cancer is the most common cancer in men and it is estimated that over 350,000 men worldwide die of prostate cancer every year. There remains an unmet clinical need to improve how clinically significant prostate cancer is diagnosed and develop new treatments for advanced disease. Aberrant glycosylation is a hallmark of cancer implicated in tumour growth, metastasis, and immune evasion. One of the key drivers of aberrant glycosylation is the dysregulated expression of glycosylation enzymes within the cancer cell. Here, we demonstrate using multiple independent clinical cohorts that the glycosyltransferase enzyme GALNT7 is upregulated in prostate cancer tissue. We show GALNT7 can identify men with prostate cancer, using urine and blood samples, with improved diagnostic accuracy than serum PSA alone. We also show that GALNT7 levels remain high in progression to castrate-resistant disease, and using in vitro and in vivo models, reveal that GALNT7 promotes prostate tumour growth. Mechanistically, GALNT7 can modify *O*-glycosylation in prostate cancer cells and correlates with cell cycle and immune signalling pathways. Our study provides a new biomarker to aid the diagnosis of clinically significant disease and cements GALNT7-mediated *O*-glycosylation as an important driver of prostate cancer progression.

## Introduction

Prostate cancer is the most common cancer in men and a major cause of cancer-related deaths [1]. The androgen receptor (AR) plays an essential role in the normal growth and development of the prostate gland, as well as in carcinogenesis [2]. The first-line treatments for advanced prostate cancer include androgen deprivation therapy (ADT), but unfortunately most tumours progress to an aggressive state for which ADT becomes ineffective, known as castration-resistant prostate cancer (CRPC). Numerous second generation agents targeting the androgen receptor signalling axis, such as abiraterone [3] and enzalutamide [4] are available for CRPC, however nearly all affected men will also develop resistance to these agents [5]. It is estimated that more than 350,000 men die of prostate cancer yearly [1], and new therapies for advanced disease are urgently needed.

Serum prostate specific antigen (PSA) measurements are often used to aid prostate cancer diagnosis, but this has poor specificity leading to the overtreatment of non-lethal disease [6, 7]. The introduction of multiparametric magnetic resonance imaging (mpMRI) has improved risk stratification for men with suspected prostate cancer [8], but there remains an unmet clinical need to identify new diagnostic and prognostic biomarkers and attention is now turning to minimally invasive urine or blood based tests [9]. The ideal liquid biopsy test would decrease over-diagnosis, allow low risk patients to avoid biopsy, increase the early diagnosis of clinically significant prostate cancer, and allow for accurate monitoring of response to therapy.

Glycosylation is the most common, complex, and dynamic post-translational modification of both membrane bound and secreted proteins [10]. Glycans are fundamental to many biological processes and hence the glycocalyx, a heavily glycosylated extramembrane compartment, is present on every mammalian cell [11]. In cancer, the size of the tumour cell glycocalyx increases with malignant transformation, and this change alters all aspects of disease progression [12–14]. Aberrant glycosylation is a hallmark of cancer cells, and cancer-associated glycans have been detected in virtually every cancer type. Glycans hold huge potential for the development of new diagnostic and therapeutic targets and are predicted to be at the forefront of translational developments. Glycan structures change dramatically in prostate cancer, and are linked to a malignant phenotype [15]. Glycan alterations in prostate cancer include changes to PSA glycosylation, tumour hypersialylation, truncated *O*-glycans and increased core fucosylation, but the mechanisms driving these changes are poorly understood [15]. One of the key drivers of aberrant glycosylation in cancer is the dysregulated expression of glycosylation enzymes within the cancer cell [16].

Previously, we identified glycosylation as an androgen-regulated process in prostate cancer and further defined a set of glycosylation enzymes that are upregulated in tumour tissue [17–20]. Among these enzymes, here we have focused on GALNT7, a glycosyltransferase transferring an *N*-acetylgalactosamine (GalNAc) moiety to Ser/Thr side chains on proteins initiating the so-called *O*-linked glycan biosynthesis [21]. GALNT7 belongs to a large family of 20 GALNT isoenzymes and is characterized for exclusively glycosylating proteins/peptides that already carry GalNAc moieties added by other GALNT-isoenzymes [22]. Recently, *GALNT7* was identified as a single gene effector of cell surface glycosylation and glycocalyx height [23], suggesting that GALNT7 mediated *O*-glycosylation might act at the interface of cellular signalling processes in prostate cancer and play an important role in disease progression.

Here, we analyse multiple independent cohorts of patient tissue samples and find upregulation of GALNT7 is a feature of prostate cancer cells. Furthermore, we show GALNT7 is found in diagnostically relevant levels in the urine and blood of men with prostate cancer. Our findings reveal urine GALNT7 can identify men with prostate cancer with improved accuracy compared to serum PSA levels, and show combining GALNT7 with serum PSA further improves diagnostic performance. Using lectin/antibody immunoassays, chemical tools and *O*-glycoproteomics, we show GALNT7 modifies *O*-glycoproteins in prostate cancer cells, and using both in vitro and in vivo studies, we further show that GALNT7 drives prostate tumour growth and correlates with cell cycle and immune signalling pathways. Our findings identify GALNT7 as an important driver of prostate cancer progression, and highlight new opportunities to exploit aberrant *O*-glycosylation to improve diagnosis and treatment.

## Results

### Upregulation of GALNT7 is a feature of prostate cancer

**Fig. 1:**
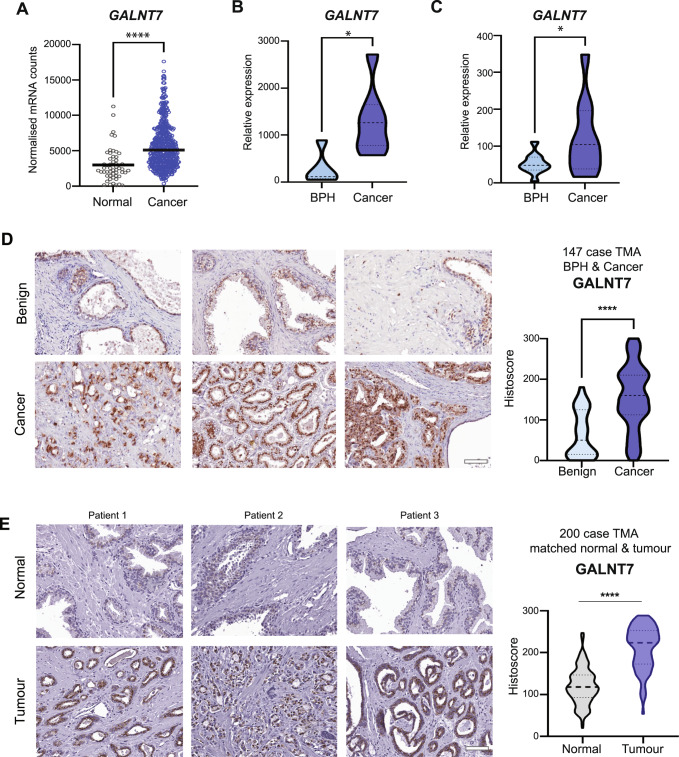
GALNT7 is upregulated in prostate cancer tissue. Analysis of GALNT7 gene and protein levels in clinical prostate tissue. **A** *GALNT7* mRNA levels in the TCGA PRAD cohort [24] are significantly higher in prostate cancer tissue relative to normal prostate tissue (*n* = 549, unpaired *t* test, *p* = <0.001). **B** Real-time PCR analysis of *GALNT7* in RNA samples extracted from FFPE prostate tissue. *GALNT7* is significantly upregulated in cancer relative to benign tissue (*n* = 12, unpaired *t* test, *p* = 0.0174). **C** Real-time PCR analysis of GALNT7 mRNA in matched normal and prostate tumour samples. *GALNT7* is upregulated in tumour tissue relative to matched normal tissue from the same patient (*n* = 20, paired *t* test, *p* = 0.0357). **D** Immunohistochemistry analysis of GALNT7 protein in a previously published prostate cancer tissue microarray (TMA) [25]. Each section was scored using the Histoscore method [77, 78]. GALNT7 is upregulated in prostate cancer tissue relative to benign prostate hyperplasia tissue (BPH) (*n* = 147, unpaired *t* test, *p* < 0.001). Scale bar is 100 µm. **E** Immunohistochemistry analysis of GALNT7 protein in a previously published TMA containing matched tumour and normal tissue from the same patient [79]. GALNT7 protein is significantly increased in prostate tumour tissue relative to matched normal tissue from the same patient (*n* = 200, paired *t* test, *p* < 0.001). Scale bar is 100 µm.

We previously reported preliminary findings suggesting that *GALNT7* gene levels are upregulated in clinical prostate cancer tissue, compared to normal or benign prostate tissue [17]. Here, we monitor GALNT7 at both the gene and protein level in multiple independent patient cohorts (comprising >2000 patient samples) and establish upregulation of GALNT7 as a feature of prostate cancer. Analysis of RNA sequencing data from The Cancer Genome Atlas Prostate Adenocarcinoma (TCGA PRAD) cohort [24] reveals *GALNT7* gene expression levels are 1.9 fold increased in prostate tumours relative to normal prostate tissue (*n* = 549, *p* < 0.001) (Fig. 1A). Using quantitative PCR, we further show upregulation of the *GALNT7* gene in prostate cancer tissue in two additional primary patient cohorts (Fig. 1B, C). To test whether GALNT7 is also upregulated at the protein level in prostate tumours, we used immunohistochemistry to monitor GALNT7 protein expression in three independent tissue microarrays (TMA). Staining a tissue microarray containing 25 samples from patients with benign disease (benign prostate hyperplasia, BPH) and 122 samples from patients with prostate cancer [25] showed that GALNT7 protein is expressed at 2.4 fold higher levels in prostate cancer tissue relative to benign tissue (*p* < 0.001) (Fig. 1D). Similarly, analysis of a TMA containing 17 normal prostate tissue samples and 79 samples of prostate tumour tissue showed GALNT7 is 2.3 fold upregulated in prostate cancer (*p* = 0.0124) (Supplementary Figure 1). Furthermore, staining of a TMA containing matched normal and prostate cancer tissue from 200 patients [26] showed GALNT7 levels were 1.8 fold higher in prostate cancer tissue compared to matched normal tissue from the same patient (*p* < 0.001) (Fig. 1E). We confirmed specificity of our GALNT7 immunohistochemistry via pre-incubation with a blocking peptide and by detection of protein depletion and overexpression by immunohistochemistry in Formalin Fixed Paraffin Embedded (FFPE) cell pellets (Supplementary Fig. 2). Taken together, our data identifies upregulation of GALNT7 as a feature of prostate tumour tissue.

### GALNT7 is upregulated in urine and blood samples from men with prostate cancer

**Fig. 2:**
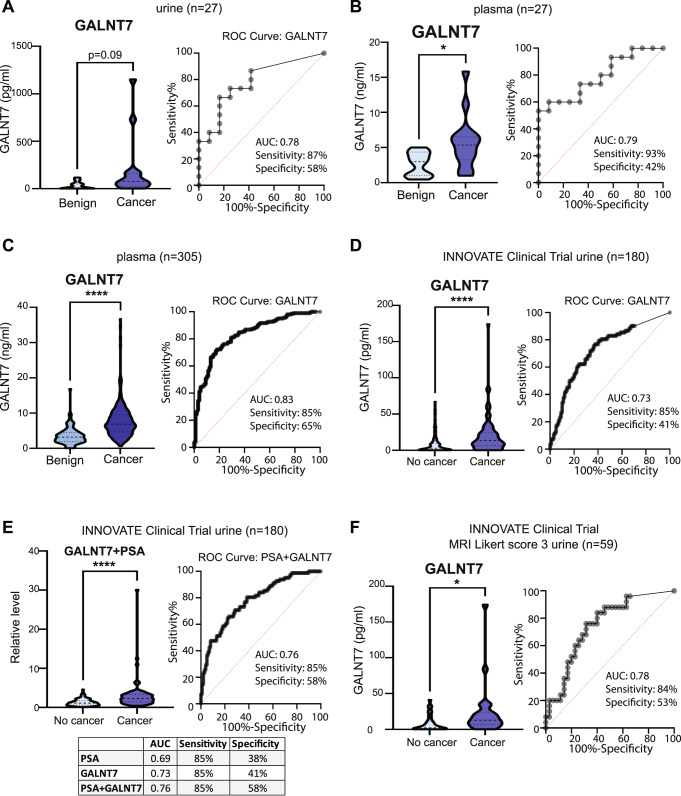
GALNT7 is upregulated in the urine and blood from men with prostate cancer. Detection of GALNT7 in patient urine and blood samples from 3 patient cohorts using pre-validated sandwich ELISA assays. **A, B** GALNT7 protein levels detected by sandwich ELISA assays in urine and matched plasma samples from men with suspected prostate cancer. Compared to men with benign disease, GALNT7 urine levels are higher in men with prostate cancer (*n* = 27, unpaired *t* test, *p* = 0.082) (AUC 0.78, sensitivity 87%, specificity 58%). Additionally, GALNT7 plasma levels are significantly higher in men with prostate cancer (*n* = 27, unpaired *t* test, *p* = 0.0172)(AUC 0.79, sensitivity 93%, specificity 42%). **C** GALNT7 levels are significantly higher in plasma samples from men with prostate cancer compared to men with benign disease (*n* = 305, unpaired *t* test, *p* < 0.0001)(AUC 0.83, sensitivity 85%, specificity 65%). **D** GALNT7 urine levels were monitored via sandwich ELISA assays in 180 men with suspected prostate cancer taking part in the INNOVATE clinical trial [31]. GALNT7 urine levels are significantly higher in men who were diagnosed with prostate cancer, compared to men who received a ‘no cancer’ diagnosis (*n* = 180, unpaired *t* test, *p* < 0.0001). GALNT7 urine level was more accurate than serum PSA at identifying men with prostate cancer (urine GALNT7 AUC: 0.73, sensitivity 85%, specificity 41%)(serum PSA AUC: sensitivity 85%, specificity 38%). **E** For the 180 men with suspected prostate cancer taking part in the INNOVATE clinical trial, the combination of serum PSA and urine GALNT7 had the greatest diagnostic power to identify those with prostate cancer (AUC 0.76, sensitivity 85%, specificity 58%). **F** For men taking part in the INNOVATE study that received mpMRI scored Likert 3, GALNT7 levels were significantly higher in men with prostate cancer (*n* = 59, unpaired *t* test, *p* = 0.0001)(AUC 0.78).

We next assessed the suitability of GALNT7 as a diagnostic biomarker in biological fluids, compared to the gold standard marker PSA. We noted that the localisation of both proteins in prostate cancer tissue was similar, which was unexpected because GALNT7 is a Golgi-resident enzyme in healthy cells while PSA is secreted into blood. Based on this, and because shedding of glycosylatransferases has been previously reported [27–30], we hypothesised that like PSA, GALNT7 may also be secreted into the urine and blood of men with prostate cancer. Using pre-validated sandwich ELISA assays (Supplementary Figure 3A), we tested GALNT7 protein levels in urine and blood samples from men with suspected prostate cancer (our power calculations showed testing at least 20 samples would give us a 90% chance of detecting any significant changes, *p* < 0.05). First, we monitored GALNT7 in matched urine and plasma samples from 27 men with suspected prostate cancer. GALNT7 levels were 7.7 fold higher in urine and 2.1 fold higher in plasma samples from men later diagnosed with prostate cancer compared to men with benign disease or a ‘no cancer’ diagnosis (Fig. 2A, B). Next, we monitored GALNT7 plasma levels in 305 men diagnosed with either benign disease or prostate cancer. GALNT7 protein levels were 2.2 fold higher in men with prostate cancer compared to men diagnosed with benign disease (*p* < 0.0001) (Fig. 2C). GALNT7 urine levels were also monitored in 180 men with suspected prostate cancer taking part in the INNOVATE clinical trial [31]. Here, urine GALNT7 was 2.5 fold higher in men diagnosed with prostate cancer (compared to men given a ‘no cancer’ diagnosis) (Fig. 2D). Urine GALNT7 had slightly improved accuracy over serum PSA at identifying men with prostate cancer within the same cohort (serum PSA: AUC 0.69, urine GALNT7: AUC 0.73), whereas combining PSA and GALNT7 further increased diagnostic accuracy (PSA + GALNT7: AUC 0.76) (Fig. 2E and Supplementary Fig. 3B).

The five-point Likert scaling on mpMRI can be used to predict clinically significant prostate cancer (Likert score 1 or 2 indicates the patient is unlikely to have prostate cancer. Likert score 4 or 5 indicates the patient is likely to have prostate cancer that needs to be treated, and with Likert score 3 it is difficult to tell from the scan if prostate cancer is present or not). Hence, for men with an equivocal mpMRI score of Likert 3 (where it isn’t possible to tell from the scan whether the patient has prostate cancer) there is an unmet clinical need for novel biomarkers to aid in risk assessment [32]. To test whether GALNT7 could improve the stratification of patients undergoing mpMRI, we evaluated GALNT7 levels in men from the INNOVATE study with mpMRI scored Likert 3 that later underwent prostate biopsy (*n* = 59). GALNT7 was 2.6 fold higher in Likert 3 patients that received a prostate cancer diagnosis compared to men given a ‘no cancer’ diagnosis (*p* = 0.0001), and GALNT7 was slightly more accurate than serum PSA at identifying men with prostate cancer (serum PSA: AUC = 0.69, urine GALNT7: AUC = 0.78) (Fig. 2F). This finding suggests urine GALNT7 levels could aid in risk assessment for patients with mpMRI scored Likert 3 and add value to the clinical care pathway. GALNT7 was also detected in urine from men with metastatic CRPC (Supplementary Fig. 3C). Taken together, our findings show that GALNT7 is upregulated in the urine and blood of men with prostate cancer and could aid in diagnosis.

### GALNT7 levels remain high in castrate resistant prostate cancer

**Fig. 3:**
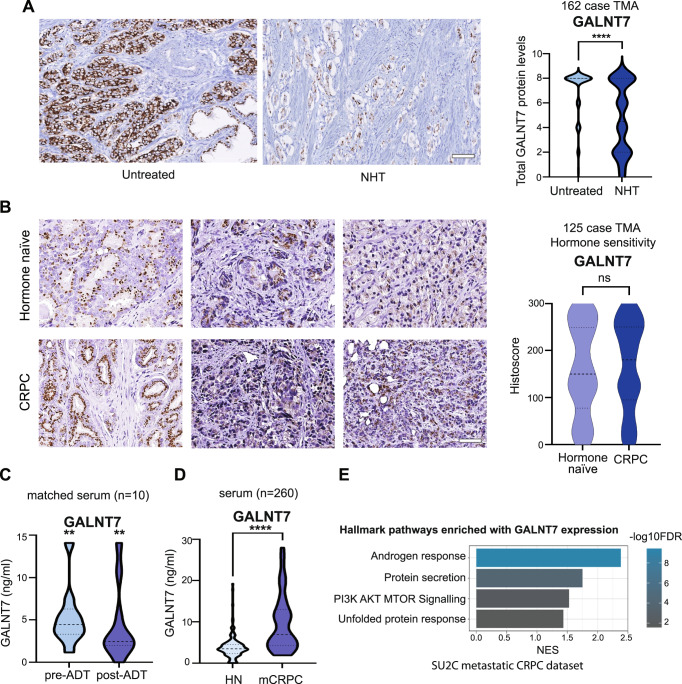
GALNT7 levels remain high in castrate resistant prostate cancer. Analysis of GALNT7 protein levels across two prostate cancer tissue microarrays (TMAs) using immunohistochemistry and in serum samples using sandwich ELISA assays. **A** Immunohistrochemistry analysis of GALNT7 levels in a previously published TMA [26]. GALNT7 levels in tumour tissue are significantly reduced after neoadjuvant hormonal therapy (NHT) (*n* = 162, unpaired *t* test, *p* < 0.0001). Scale bar is 100 µm. **B** Analysis of a 125 case TMA reveals the levels of GALNT7 in hormone naïve and castrate resistant prostate cancer (CRPC) are similar. Scale bar is 100 µm. **C** GALNT7 serum levels significantly decrease following ADT (*n* = 20, Mann Whitney U test, *p* = 0.0039). **D** The serum levels of GALNT7 in men with mCRPC are significantly higher than in men with hormone naïve prostate cancer (*n* = 260, unpaired *t* test, *p* < 0.001). **E** In the SU2C mCRPC patient cohort (*n* = 163) [33, 80] GALNT7 mRNA levels correlate with the androgen response pathway (NES 2.45; FDR *q* value = <0.001).

Androgen hormones play a key role in the progression and treatment of prostate cancer and ADT is a first-line treatment used to control cancer growth in cases of metastatic prostate cancer [2]. We previously showed that expression of *GALNT7* is controlled by the AR and that GALNT7 is upregulated in prostate cancer cells in response to androgen stimulation [17]. Here, experimental analyses of gene expression data in The Cancer Genome Atlas Prostate Adenocarcinoma (TCGA PRAD) [24] and the metastatic CRPC Stand Up to Cancer/Prostate Cancer Foundation (SU2C/PCF) [33] cohorts show a significant correlation between the expression of *AR* and *GALNT7* in both clinical prostate cancer datasets (Supplementary Fig. 4A, B). Further experimental analyses reveal *GALNT7* mRNA is downregulated in prostate tissue following androgen depletion (Supplementary Fig. 4C, D) [34–36]. Based on these findings, we hypothesised that GALNT7 protein levels in prostate tissue will decrease in patients undergoing ADT. To test this, we monitored GALNT7 protein levels in a TMA containing untreated prostate tissue and tissue samples taken 3-12 months after ADT (*n* = 162) [26]. Consistent with GALNT7 being controlled by androgens, our findings show GALNT7 protein levels are 1.5 fold lower in prostate tissue following ADT, compared to untreated tissue (*p* = 0.001) (Fig. 3A). Next, to gain insight into how GALNT7 is expressed during prostate cancer progression, we analysed GALNT7 in a TMA with hormone sensitive and CRPC patients (*n* = 125). Here, GALNT7 levels were similar in CRPC compared to hormone sensitive disease, which would be consistent with AR reactivation in castrate resistant disease (Fig. 3B). We then set out to test if serum levels of GALNT7 might also change upon ADT. In matched serum samples from ten men taken before and after hormone ablation therapy, serum GALNT7 was significantly reduced following ADT (*p* = 0.0039) (Fig. 3C). Furthermore, GALNT7 levels were significantly higher in sera from patients with metastatic CRPC relative to patients with hormone sensitive disease (*p* < 0.001) (Fig. 3D). Finally, analysis of *GALNT7* gene expression in the SU2C/PCF mCRPC tissue cohort [33] identified a significant correlation between GALNT7 and androgen receptor signalling in metastatic CRPC patient samples (*n* = 163, FDR q value <0.001)(Fig. 3E and Supplementary Table 1). Of particular interest, significant correlations were observed between *GALNT7* and *CAMKK2*, *TMPRSS2*, *CCND1* and *FKBP5*. These results indicate that GALNT7 levels directly correlate with AR activity in prostate cancer and suggest GALNT7 levels may mark those developing relapse to castrate resistant disease.

### GALNT7 can modify *O*-glycosylation in prostate cancer cells

**Fig. 4:**
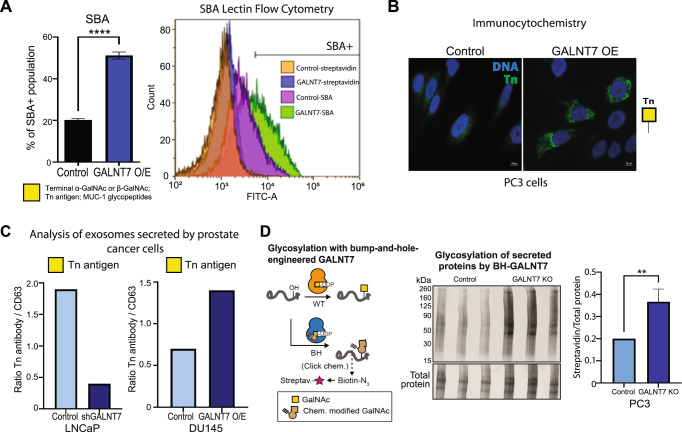
GALNT7 can modify *O*-glycosylation in prostate cancer cells. **A** Lectin flow cytometry shows PC3 cells with upregulated GALNT7 have increased binding to SBA lectin (which recognises terminal GalNAc or Tn antigen). **B** Detection of the cancer-associated Tn antigen by immunofluorescence in DU145 cells show upregulation of GALNT7 promotes increased expression of the Tn antigen. **C** Analysis of extracellular vesicles (EVs) in conditioned media from LNCaP and DU145 cells with knockdown or overexpression of GALNT7 suggests a correlation between GALNT7 and the Tn antigen in the prostate cancer secretome. **D** Detection of glycosylation of secreted proteins in samples from PC3 control cells or GALNT7-KO cells using GALNT7 bump-and-hole engineering. The active site of GALNT7 was engineered by mutation (IIe and Leu to 2xAla). The mutant (termed BH) uses a chemically modified analogue of the native substrate UDP-GalNAc. Following glycosylation, the chemical modification can be traced by methods of click chemistry.

*GALNT7* was recently identified as a single gene effector of cell surface glycosylation [23]. However, how GALNT7 impacts glycosylation in prostate cancer cells has not been previously studied. GALNT enzymes are responsible for the initiation of GalNAc *O*-linked glycosylation and the synthesis of α-GalNAc1,3-*O*-Ser/Thr, or the Tn antigen. This structure is normally extended by the further sequential action of glycosyltransferases to build more complex *O*-linked structures, but in cancer the Tn antigen is often left unelaborated, and its presence is associated with poor patient prognosis [37]. Based on this, we hypothesised that upregulation of GALNT7 in prostate cancer cells would alter *O*-linked glycosylation, including exposure of truncated *O*-glycans such as the cancer-associated Tn antigen. To test if upregulation of GALNT7 can alter the cell surface *O*-glycosylation of prostate cancer cells, we created and validated stable prostate cancer cell line models with knockdown or up-regulation of GALNT7 (Supplementary Figure 5) and assessed the recognition by lectins. Lectin arrays and flow cytometry revealed that GALNT7 overexpression correlates with increased binding of SBA lectin (which recognises the cancer-associated Tn antigen [38, 39]) (Fig. 4A and Supplementary Fig. 6). This finding was confirmed via immunocytochemistry using an antibody specific to the Tn antigen (Fig. 4B), indicating that upregulation of GALNT7 introduces tumour-associated glycans on prostate cancer cells.

**Fig. 5:**
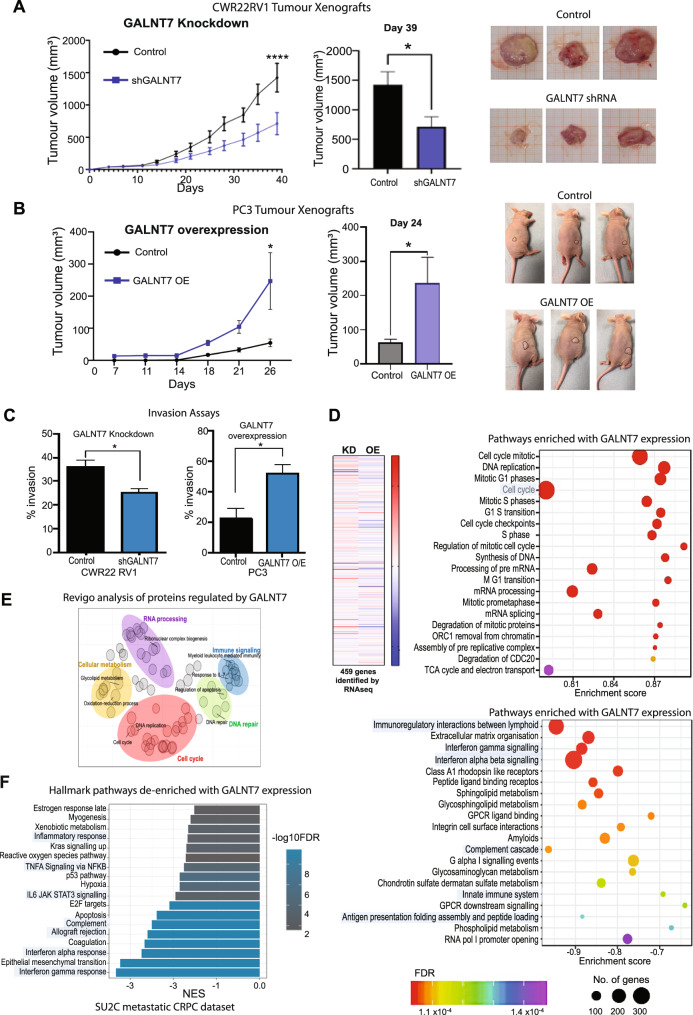
GALNT7 promotes prostate tumour growth and correlates with cell cycle and immune signalling pathways in prostate cancer cells. **A** Knockdown of GALNT7 using shRNA significantly reduces the growth of CWR22RV1 tumours xenografts in a subcutaneous xenograft model. **B** Upregulation of GALNT7 in PC3 cells significantly increases the growth of subcutaneous xenograft tumours. **C** Knockdown of GALNT7 decreases prostate cancer cell invasion and upregulation of GALNT7 promotes prostate cancer cell invasion. **D** RNAseq analysis of CWR22RV1 cells with knockdown of GALNT7 and DU145 cells with upregulated GALNT7 identified 457 genes that dynamically change in response to GALNT7. Gene Ontology analysis of the 457 genes regulated by GALNT7 shows an enrichment of genes with roles in the ‘cell cycle’ and a de-enrichment of genes with roles in ‘immune signalling’. **E** Proteomics analysis of DU145 cells with upregulation of GALNT7 identified 249 proteins with a significant change in expression levels. Revigo analysis of these 249 proteins identified ‘cell cycle’ and ‘immune signalling’ as significantly enriched pathways. **F** Analysis of the SU2C mCRPC cohort [33] identified immune related pathways (including interferon gamma response, interferon alpha response, complement, allograft rejection, IL6 signalling, TNFA Signaling via NFKB and inflammatory response) with attenuated enrichment with *GALNT7* expression.

As over 80% of the secretome is *O*-glycosylated [40], we predicted that upregulation of GALNT7 will also alter the secretome of prostate cancer cells. To investigate GALNT7-mediated *O*-glycosylation in the secretory pathway, we analysed conditioned media samples using lectin/antibody immunoassays, an implemented GALNT7 chemical biology reporter system [41], and *O*-glycoproteomics. Lectin/antibody profiling of extracellular vesicles (EVs), isolated from conditioned media, revealed increased levels of the Tn antigen when GALNT7 is upregulated, and reduced levels of Tn antigen when GALNT7 is downregulated (Fig. 5C and Supplementary Figure 7). Next, we established a GALNT7 chemical biology reporter system for the activity of the GALNT7 enzyme based on a tactic termed ‘bump-and-hole engineering’. Breifly, we engineered GALNT7 by mutation such that the resulting mutant (‘BH’ for bump-and-hole) recognises a chemically modified version of the activated sugar UDP-GalNAc [41, 42] (Fig. 4D). Following glycosylation by BH-GALNT7, the chemical modification can then be reacted with a biotin reporter probe using click chemistry [41–44]. Using the BH-GALNT7 reporter, we report specifically GALNT7 dependent glycosylation of secreted proteins in prostate cancer cells (Fig. 4D). Next, we analysed the *O*-glycoproteome in secretomes of DU145 prostate cancer cells with upregulated GALNT7 using mass spectrometry. This identified 34 glycopeptides as potential substrates for the GALNT7 enzyme in prostate cancer cells, the Tn antigen being the most abundant *O*-glycosylated form (Supplementary Figure 8). Taken together, our findings reveal that in prostate cancer cells, GALNT7 can modify the *O*-glycosylation of both cell surface proteins and proteins in the secretory pathway, leading to upregulation of the Tn antigen, which is a hallmark of cancer and involved in tumor progression and metastasis [38].

### GALNT7 promotes prostate tumour growth and correlates with cell cycle and immune signalling pathways

We next investigated the effects of GALNT7 on the biology of prostate cancer cells showing that knockdown of GALNT7 inhibits prostate cancer cell proliferation and colony formation in vitro, whereas overexpression of GALNT7 has the opposite effect (Supplementary Fig. 9A–D). Using sub-cutaneous in vivo mouse models, we found that knockdown of GALNT7 significantly suppressed the growth of CWR22RV1 tumours (Fig. 5A), whereas overexpression of GALNT7 significantly increased the growth of PC3 tumours (Fig. 5B). Furthermore, using in vitro assays we show GALNT7 promotes prostate cancer cell migration and invasion (Fig. 5C and Supplementary Fig. 9E–G). Next, we analysed the effects of conditioned media on prostate cancer cell biology. These experiments showed that conditioned media from prostate cancer cells with upregulated GALNT7 significantly enhanced cell proliferation and colony formation of wildtype prostate cancer cells, indicating that this effect was likely mediated by the secreted GALNT7, other unknown proteins of the media or others molecules (Supplementary Fig. 9H, I). Taken together, the above data suggest that upregulation of GALNT7 promotes aggressive prostate cancer cell behaviour and tumour growth.

To identify signalling pathways controlled by GALNT7 in prostate cancer cells, we used RNA-sequencing to search for genes that change with either knockdown or upregulation of GALNT7. Bioinformatic analyses identified 457 genes that respond to GALNT7 levels (Supplementary Tables 2–4 and Supplementary Fig. 10A, B) with enrichment in the cell cycle processes and depletion in immune signalling pathways (Fig. 5D and Supplementary Fig. 10C). By analysing the proteome of GALNT7 overexpressing cells using mass spectrometry we identified 249 differentially expressed proteins, and also highlighted ‘cell cycle’ and ‘immune signalling’ as significantly altered by GALNT7 (Fig. 5E and Supplementary Table 5). Of particular interest, expression levels of the tumour suppressor gene *FOXO1* were increased upon knockdown of GALNT7, and decreased when GALNT7 is overexpressed. FOXO1 is an important negative regulator of the cell cycle [45] that is often lost or downregulated in prostate cancer [46]. Validation at the protein level confirmed that upregulation of GALNT7 in prostate cancer cells promotes loss of FOXO1 protein (Supplementary Fig. 10D). Analysis of The Cancer Genome Atlas Prostate Adenocarcinoma (TCGA PRAD) cohort [24] revealed a significant correlation between the *GALNT7* and *FOXO1* genes in clinical prostate cancer tissue (Supplementary Fig. 10E). Furthermore, analysis of the metastatic CRPC Stand Up to Cancer (SU2C) cohort [33] identified seven ‘immune signalling’ pathways which negatively correlate with *GALNT7* gene expression levels in clinical samples (Fig. 5F and Supplementary Table 1).

## Discussion

Prostate cancer is the most common cancer in men and is a major clinical burden. New diagnostic tests and therapeutic options are urgently needed and could aid in patient stratification and improve patient quality of life and survival times. In prostate cancer, aberrant glycosylation is closely linked to a malignant phenotype, however the mechanisms driving these changes are unclear. An increased understanding of how altered glycosylation contributes to prostate cancer pathology will uncover an untapped resource of biomarkers and therapeutic targets, and has the potential to transform how the disease is diagnosed and treated.

In this study, we measured levels of the glycosylation enzyme GALNT7 in prostate cancer tissue, urine and blood samples and identified GALNT7 as being significantly upregulated in prostate cancer. By analysing tumour and blood samples from men with prostate cancer, we further show that GALNT7 levels remain high in castrate resistant prostate cancer, thus suggesting GALNT7 is increased during both the development of prostate cancer and the progression to relapsed treatment resistant disease. Traditionally, the diagnosis of prostate cancer has relied on the detection of prostate specific antigen (PSA) in patient blood, and tissue biopsy tests - however this is now widely accepted as being sub-optimal [8, 47]. The introduction of pre-biopsy mpMRI (multiparametric magnetic resonance imaging) has improved risk stratification for men with suspected prostate cancer [8, 48, 49], but there remains space for novel serum and urine biomarkers in this new pathway. In particular, many patients with Likert 3 disease on mpMRI still undergo biopsy but do not have clinically significant prostate cancer [32]. Here, we demonstrate urine GALNT7 can identify men with prostate cancer with improved accuracy than serum PSA levels, and show that combining GALNT7 with PSA further improves diagnostic performance. Furthermore, in mpMRI Likert 3 patients, we show urine GALNT7 holds promise to identify patients with clinically significant disease. Based on these data, we propose that GALNT7 is upregulated in prostate cancer and could be developed further as a as a non-invasive diagnostic biomarker.

Our findings also show GALNT7 can modify the *O*-glycosylation of both cell surface proteins and proteins in the secretory pathway, and identify GALNT7 as a key driver of tumour growth which correlates with cell cycle and immune signalling pathways in prostate cancer cells. These data are consistent with the recent finding that *GALNT7* is a single gene effector of cell surface glycosylation and glycocalyx height [23], and suggest GALNT7 mediated *O*-glycosylation may act at the interface of important signalling pathways in prostate cancer cells to promote disease progression. Based on these findings, we propose GALNT7 is a key player in prostate cancer that can likely be exploited for therapeutic usage. Targeting aberrant glycosylation holds huge potential for cancer research [50–52], and we envisage that both GALNT7, and its associated glycans (particularly the cancer-associated Tn antigen) hold huge promise in prostate cancer therapy that needs to be explored.

Strategies to target glycosylation in cancer include, the use of carbohydrate analogues, the development of glycan specific chimeric antigen receptor (CAR) T, and the discovery of small molecule inhibitors with potential clinical applications [50]. Glycosyltransferase enzymes such as GALNT7 have the potential to be used for the discovery of effective cancer drugs. Specific inhibitors of GALNT enzymes are lacking, but an isozyme-selective inhibitor targeting GALNT3 was recently developed and shown to block the invasion of breast cancer cells with no toxic effects [53], thus paving the way for the discovery of small molecule modulators that control the activity of specific GALNTs. Moving forward, we anticipate the development of both isozyme and pan-specific modulators targeting GALNT7 will yield new inhibitors, conceptually related to the widespread use of drugs targeting protein kinases, to provide a new class of therapeutics to treat prostate cancer.

The data presented indicates upregulation of GALNT7 may increase the expression of the Tn antigen in prostate cancer. Tn is an important tumour-associated glycan and an established druggable target for several cancer types [15, 32, 33]. Studies show the Tn antigen is limited to cancers and is not expressed in healthy/normal tissue [54, 55]. Potential strategies to target Tn for cancer therapy include monoclonal antibodies [56], therapeutic vaccination [57–59], and engineered CAR T cells against Tn antigen on MUC1 [60]. Emerging research shows the Tn antigen contributes to an immune suppressive microenvironment and is a promising target for immunotherapy [32, 61–64]. Consistent with this, our findings indicate GALNT7 correlates with immune signalling pathways in prostate cancer. As Tn expression has been detected in up to 90% of prostate tumours [58, 65–67] there is huge potential to exploit this biology to benefit patients.

The absence of a glycosylation consensus sequence and the variability of glycan elaboration have historically made *O*-GalNAc glycans challenging to study. Hence, little is known about the protein substrate specificity of GALNT7, other than its requirement for previously *O*-glycosylated substrates [21, 68]. Here, we equipped cells with the ability to tag protein substrates of GALNT7 and report GALNT7 dependent glycosylation of secreted proteins in living prostate cancer cells. Alongside this, we performed site specific *O*-glycoproteomics to identify 34 potential protein substrates for GALNT7. It is tempting to speculate that the enhancement of prostate cancer cell proliferation by GALNT7 is likely due to either the activity of secreted GALNT7, or some of its protein substrates’ roles or combined functions. Taken together, our findings suggest that further characterisation of GALNT7 mediated *O*-glycosylation, alongside a systematic study of *O*-glycosylation in prostate tumours, will reveal novel biomarkers and potential therapeutic targets to provide clinically actionable information that could impact men with prostate cancer.

## Methods

### Cell culture and creation of stable cell lines

Cell culture and the cell lines used were as described previously [69]. The stable cell lines used in the study were created by lentiviral transduction using an MOI of 5. For details of the lentiviral particles used please see supplementary table 6. To generate GALNT7 KO cells guide RNAs ACATGAGGCCATGGTACCAC and GTACCATGGCCTCATGTTGA were cloned into the LentiCRISPRv2 plasmid (Addgene plasmid 52961). Lentivirus production, transduction and selection of monoclonal cell lines was carried out as previously described [70]. Cell lines were obtained from ATCC, authenticated using DNA STR analysis, and tested every 3 months for mycoplasma contamination.

### Western blotting

Western blotting was performed as previously described [69]. For details of the antibodies used please see Supplementary Table 6.

### Quantitative PCR

Quantitative PCR (qPCR) was performed as previously described [69]. For details of the primers used please see Supplementary Table 6.

### Immunohistochemistry

For details of the TMAs tested and methods used please see Supplementary Table 7.

### Detection of GALNT7 in urine and serum

Human GALNT7 sandwich pre-validated ELISA kits were purchased from Cambridge Bioscience (RayBioTech, ELH-GALNT7-1). Samples and standards were assayed in duplicate according to the manufacturer’s protocol. For specific details of the methods used for each cohort please see see Supplementary Table 8.

### In vitro cell behavior assays

Cell proliferation assays were carried out using the WST-1 96 well Cell Proliferation Assay Kit (Cambridge Bioscience, CAY10008883) as per the manufacturer’s instructions. Colony formation assays were performed as previously described [19]. Invasion assays were carried out using the Oris Pro 96-well Invasion Assay (Amsbio, PROIA1) as per the manufacturer’s instructions.

### Mouse models

#### CWR22RV1 tumour xenografts

Male NMRI mice (Charles Rivers) were inoculated at 8 weeks of age with 1 × 10^7^ CWR22RV1 cells with GALNT7 knockdown by unilateral subcutaneous injection into the flank. The mice were randomised into control or treatment groups before cancer cell inoculation. Cells were injected in a volume of 100 µL cell culture media and Matrigel in a 1:1 mixture. Animals were weighed and tumour volumes monitored by caliper measurement three times a week by a blinded researcher until the first animal met a humane endpoint (defined as tumor volume reaching 2000 mm^3^). Tumors with ulceration were excluded from the analysis.

#### PC3 tumour xenografts

Male CD-1 Nude mice (Charles Rivers) were inoculated at 8 weeks of age with 1 × 10^7^ PC3 cells with GALNT7 overexpression. The mice were randomised into control or treatment groups before cancer cell inoculation. Cells were injected in a volume of 50 µL of cell culture media and Matrigel in a 1:1 mixture. Animals were weighed and tumour volumes monitored by caliper measurement three times a week by an unblinded researcher until the first animal met a humane endpoint (defined as tumor volume reaching 1000 mm^3^). Tumors with ulceration were excluded from the analysis. Sample size estimate was based on our recent experience of similar xenograft studies. All animal work was conducted with project license approval granted by the UK Home Office and under the approval of the local biomedical research ethics committee.

### Lectin array and lectin flow cytometry

The glycosylation profile of PC3 cells with upregulation of GALNT7 was monitored using a 26 lectin microarray kit (Z Biotech, 10606-K) according to the manufacturer’s instructions. Lectin flow cytometry was performed as described previously, using biotinylated SBA lectin (Vector labs, B-1015-5).

### Isolation and analysis of Extracellular Vesicles

Detection of glycosylation changes of extracellular vesicles (EVs) from cell culture media was performed using a previously published nanoparticle based time-resolved fluorescence immunoassay (NP-TRFIA) [71] (further details are provided in Supplementary Fig. 8). The effect of GALNT7 KD or OE on glycosylations on EVs was calculated based on the signal-to-background (S/B) levels of Tn-assays. The CD63-CD63 signals were used to quantify the EV amount in each cell line.

### GALNT7 bump-and-hole engineering and bioorthogonal labelling of secretome proteins by bump-and-hole engineered GALNT7

Bump- and-hole engineering of GALNT7 in living cells was performed based on previously established protocols [41]. For further details of the methods used see Supplementary Table 9.

### Mass spectrometry-based proteomics and glycoproteomics

Mass spectrometry proteomics and glycoproteomics methods were adapted from previously described protocols [72–74]; for more details see Supplementary Table 10.

### RNAseq and GO analysis

The RNA sequencing (RNA-seq) of cell lines was performed as described previously [19] and can be accessed on GEO repository (GSE220942). Data were further analysed using the gene ontology resource (http://geneontology.org) (accessed on 3 June 2020) [75, 76]. For details of how the SU2C/PCF mCRPC tissue cohort was analysed please see Supplementary Table 11.

### Statistical analyses

All statistical analyses were performed using GraphPad Prisms 8 (GraphPad Software, Inc., San Diego, CA, USA). Data are presented as the mean of three independent samples ± standard deviation of the mean (SD). Unless otherwise stated, the variance between groups being statistically compared was similar. Statistical significance is indicated as *p < 0.05, **p < 0.01, ***p < 0.001 and ****p < 0.0001.

## Supplementary information

Supplementary Figure legends

Supplementary Figure 1

Supplementary Figure 2

Supplementary Figure 3

Supplementary Figure 4

Supplementary Figure 5

Supplementary Figure 6

Supplementary Figure 7

Supplementary Figure 8

Supplementary Figure 9

Supplementary Figure 10

Supplmentary Tables

## Data Availability

The datasets generated during and analysed during this study are available in the figshare data repository. 10.6084/m9.figshare.21185902.
