# Supplementary material for: Upregulation of GALNT7 in prostate cancer modifies O-glycosylation and promotes tumour growth
Source: Oncogene. Author manuscript; Available in PMC 2023 Mar 20. (PMC10020086; doi:10.1038/s41388-023-02604-x)

Supplementary Figure 1
GALNT7 is upregulated in prostate cancer tissue compared to normal prostate tissue

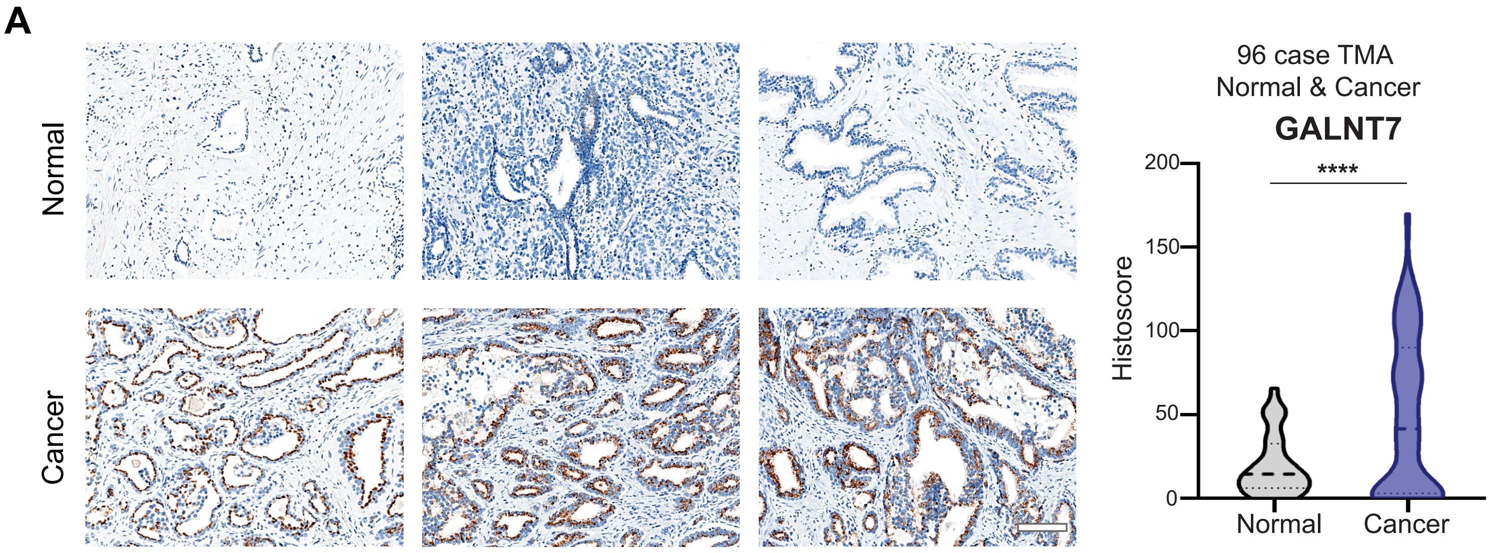

Supplement: Supplementary Figure 1 [file EMS162589-supplement-Supplementary_Figure_1.pdf]
