# Supplementary material for: Upregulation of GALNT7 in prostate cancer modifies O-glycosylation and promotes tumour growth
Source: Oncogene. Author manuscript; Available in PMC 2023 Mar 20. (PMC10020086; doi:10.1038/s41388-023-02604-x)

Supplementary Figure 2
Validation of GALNT7 by immunohistochemistry (GALNT7 antibody used at 1:1000)

A. Blocking with GALNT7 immunising peptide

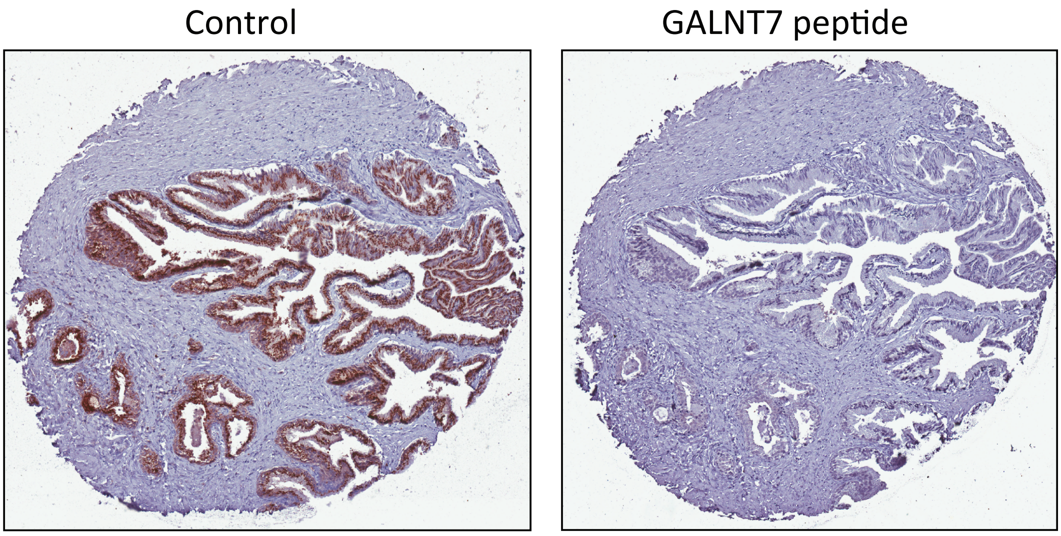

B. Immunohistochemistry staining of FFPE cell pellets depleted of GALNT7 using siRNA

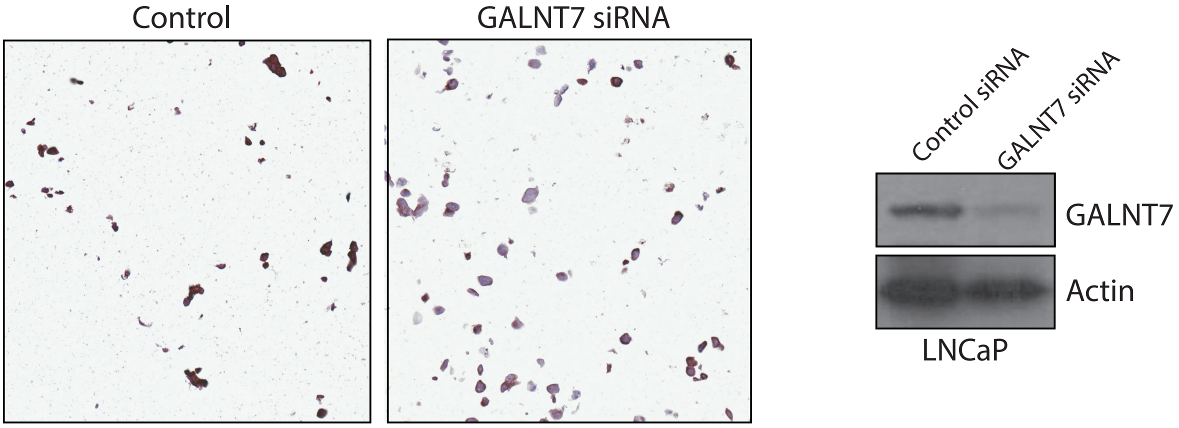

C. Immunohistochemistry staining of FFPE cell pellets with GALNT7 overexpression

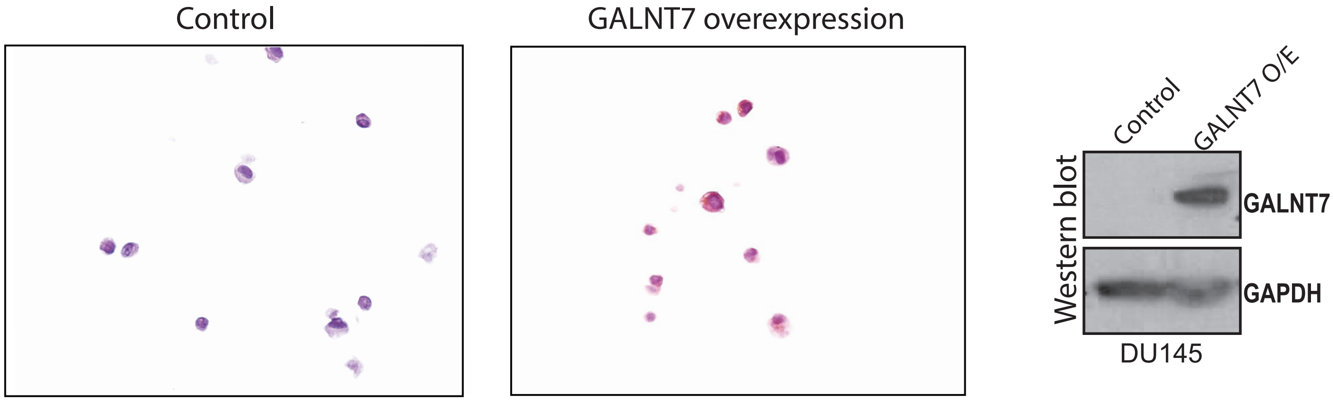

Supplement: Supplementary Figure 2 [file EMS162589-supplement-Supplementary_Figure_2.pdf]
