# Supplementary figures and images for: Upregulation of GALNT7 in prostate cancer modifies O-glycosylation and promotes tumour growth
Source: Oncogene. Author manuscript; Available in PMC 2023 Mar 20. (PMC10020086; doi:10.1038/s41388-023-02604-x)

Supplementary Figure 3

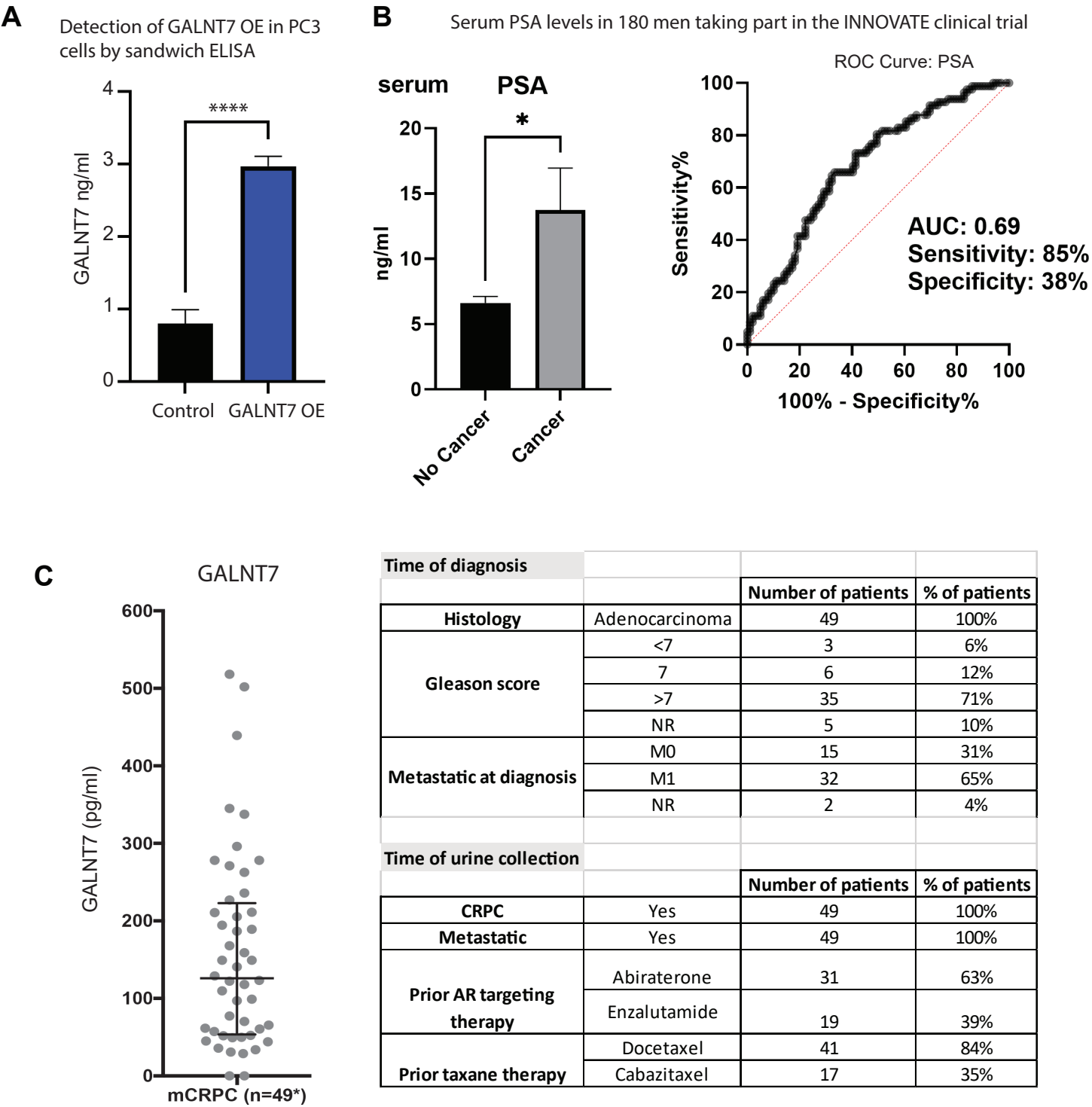

Supplement: Supplementary Figure 3 [file EMS162589-supplement-Supplementary_Figure_3.pdf]

Supplementary Figure 4  
GALNT7 is controlled by androgens in prostate cancer cells

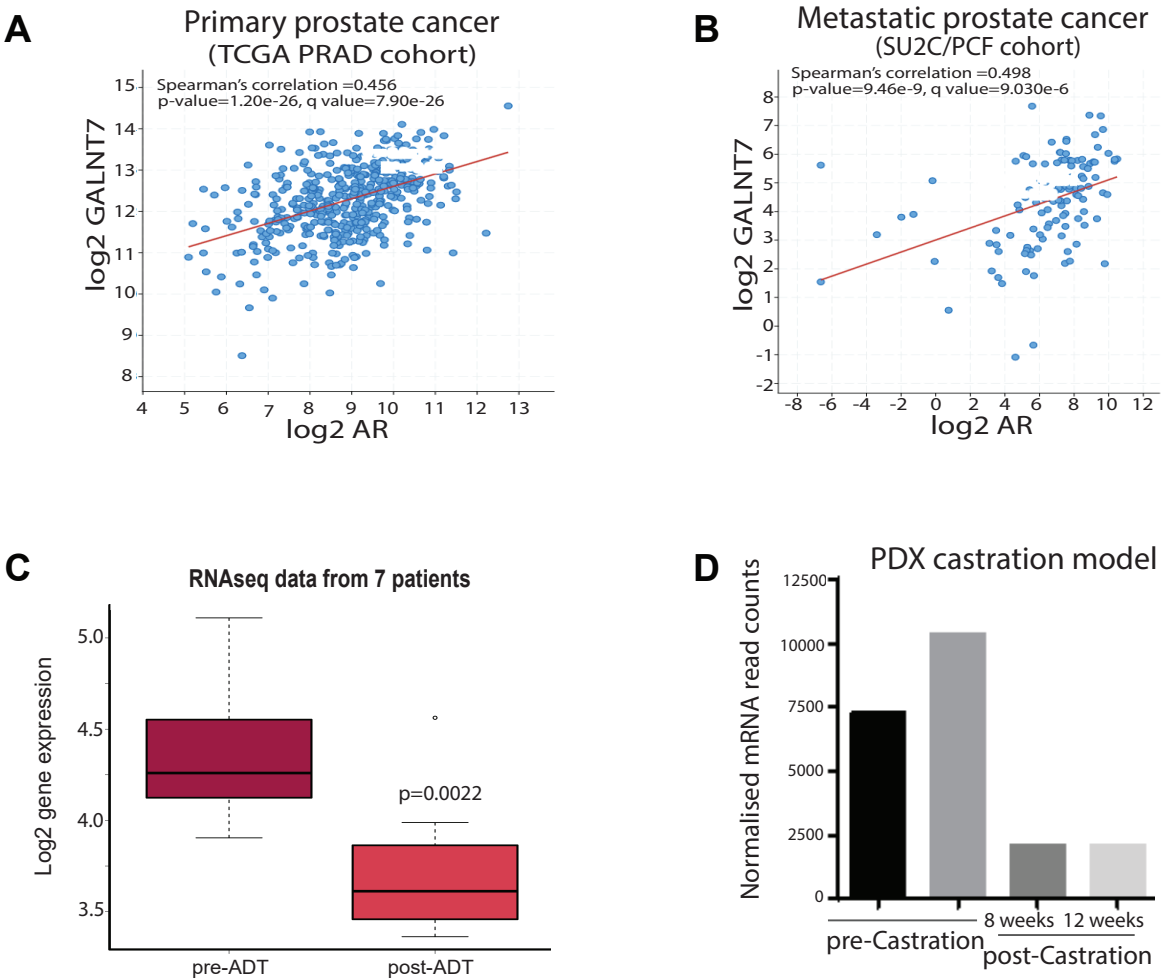

Supplement: Supplementary Figure 4 [file EMS162589-supplement-Supplementary_Figure_4.pdf]

Supplementary Figure 6  
Lectin microarray analysis of prostate cancer cells

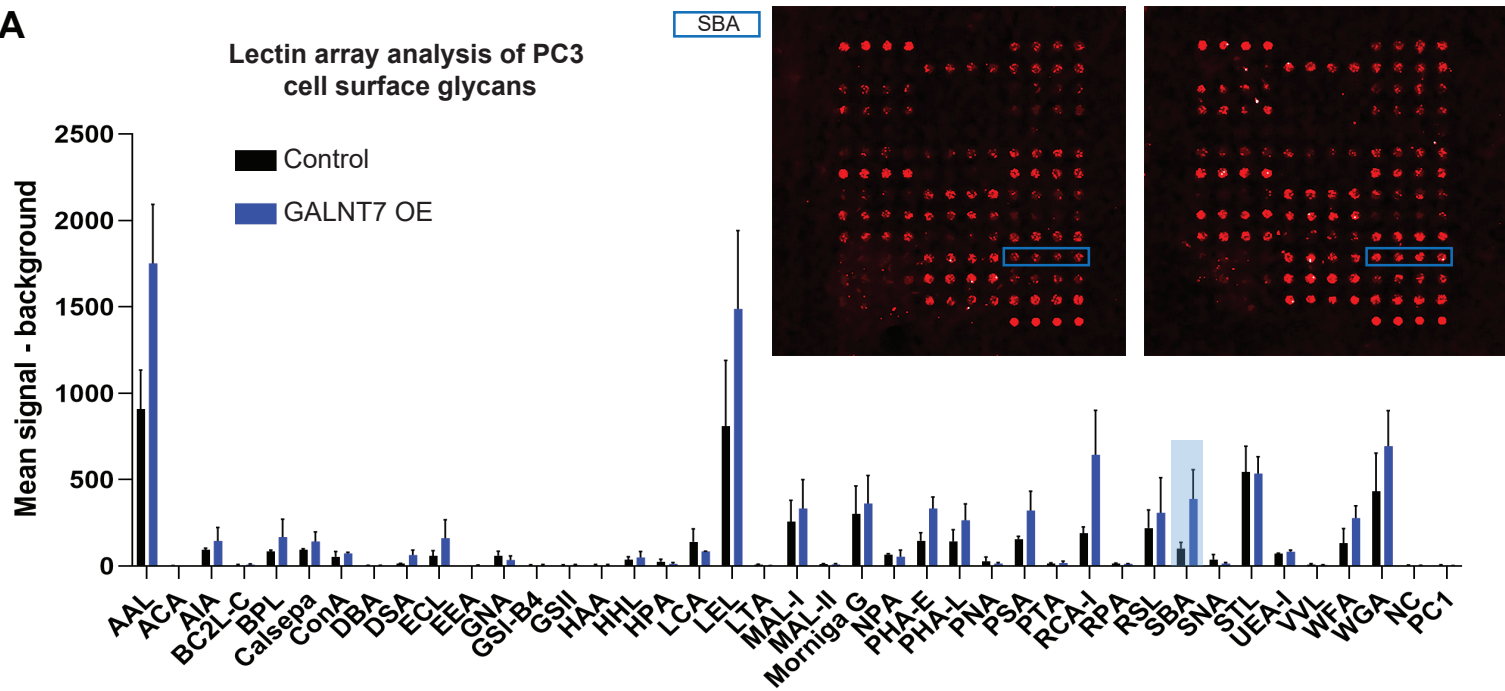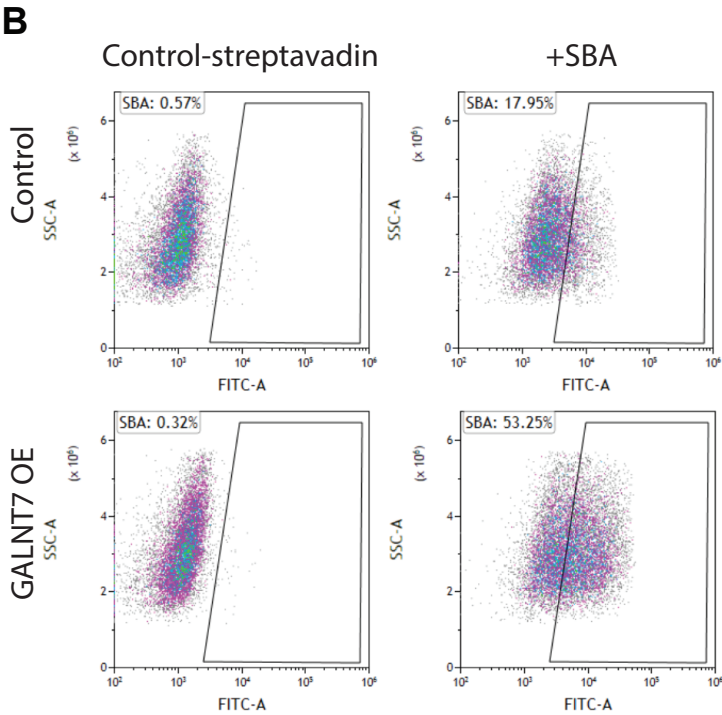

Supplement: Supplementary Figure 6 [file EMS162589-supplement-Supplementary_Figure_6.pdf]
