# Supplementary material for: Upregulation of GALNT7 in prostate cancer modifies O-glycosylation and promotes tumour growth
Source: Oncogene. Author manuscript; Available in PMC 2023 Mar 20. (PMC10020086; doi:10.1038/s41388-023-02604-x)
Supplement: Supplementary Figure 7 [file EMS162589-supplement-Supplementary_Figure_7.pdf]

Supplementary Figure 7

Lectin and antibody profiling of prostate cancer extracellular vesicles

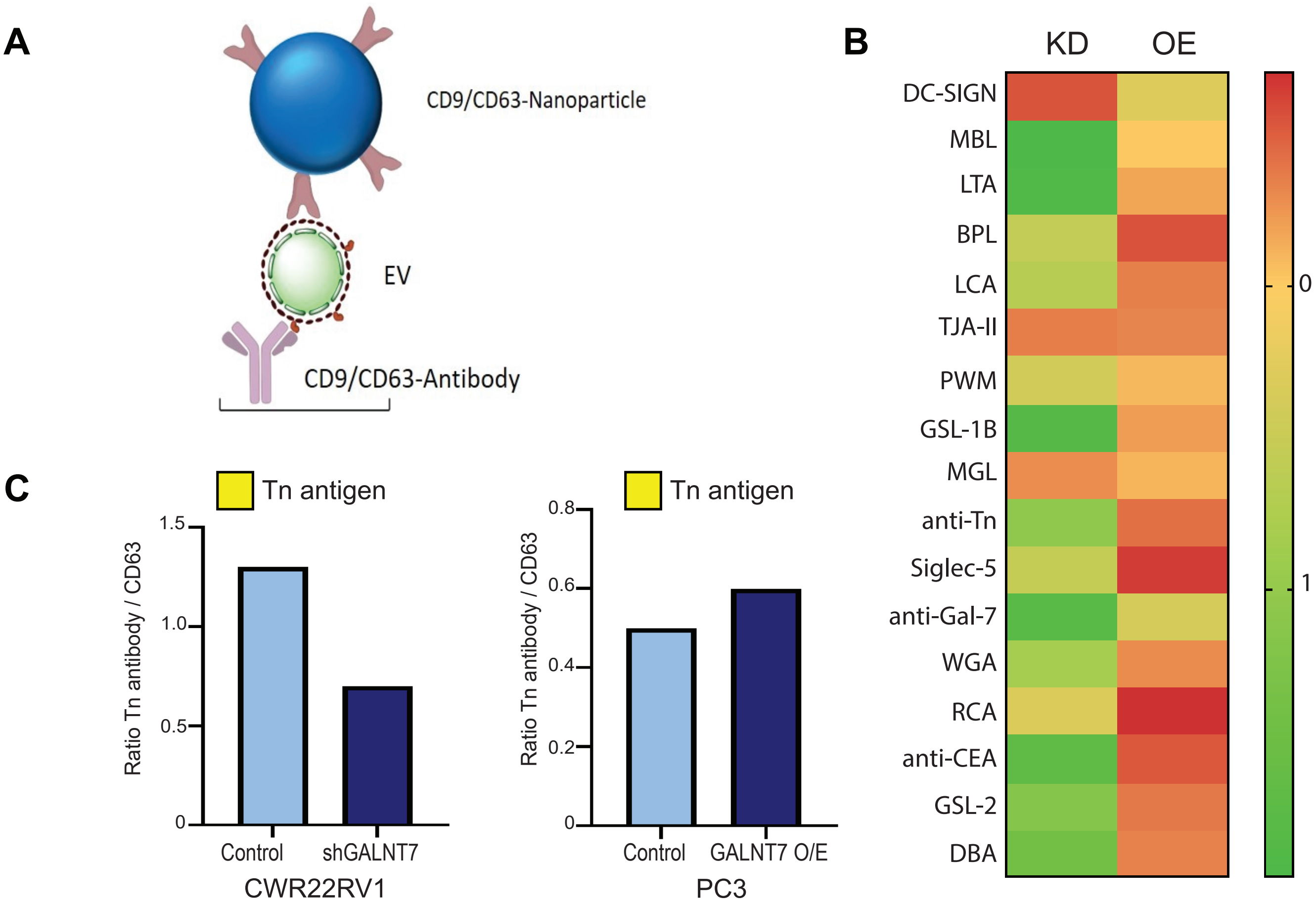

Methods for isolation and analysis of Extracellular Vesicles

Streptavidin (SA) coated low-fluorescence microtitration plates, wash buffer, and the assay buffer was purchased from Kaivogen Oy (Turku, Finland). Tetraspanin specific antibody, mAb-CD63 (clone 556019, BD Bioscience) was used to capture EVs from the conditioned cell culture media. The captured EVs were detected with europium (III)-doped Fluoro-Max polystyrene nanoparticles (Eu3+-NPs; 95 nm in diameter, Seradyn Inc., Indianapolis, IN) coated with CD63 and Tn specific antibodies (kindly provided by Fujirebio Diagnostics), MGL lectin (4888-CL-050, R&D Systems) and SBA lectin (B-1015-5, Vector laboratories).

Biotinylated anti-CD63 antibody was diluted in RED assay buffer (150 ng/30 µL/well) and immobilized on streptavidin-coated 96-well plate. The wells were washed 2 times with wash buffer before adding of cell culture medium (50 µL/well) diluted 1:2 in RED assay buffer. After two washes CD63, Tn, MGL or SBA coated nanoparticles were added (30 µL/well) to the wells and incubated 90 min. After six washes, time-resolved europium fluorescence (λex: 340 nm; λex: 615 nm) was measured from the surface of the wells with a VictorTM 1420 multilabel counter (Perkin-Elmer). All assay steps were performed at room temperature.
