# Supplementary material for: Upregulation of GALNT7 in prostate cancer modifies O-glycosylation and promotes tumour growth
Source: Oncogene. Author manuscript; Available in PMC 2023 Mar 20. (PMC10020086; doi:10.1038/s41388-023-02604-x)

Supplementary Figure 8

O-glycoproteomic analysis of DU145 prostate cancer cells with upregulated GALNT7

Gene Name	Glycan Position	Glycan	p-value
NPM1	S_{143}, S_{149}	Glycan 9, Glycan 8	**
TARS2	T_{172}, S_{176}	Glycan 6, Glycan 9	**
IGFBP6	S_{120} or T_{126}	Glycan 6	***
HRNR	S_{654},S_{656}	Glycan 6, Glycan 2	**
EFNA1	T_{152} or S_{154}	Glycan 6	***
CSF1	T_{363},T_{365}	Glycan 8, Glycan 5	**
CSF1	T_{363},T_{365}	Glycan 6, Glycan 6	**
MUC16	S_{2842},T_{2843}	Glycan 4, Glycan 7	**
PSAP	S_{216},T_{217}	Glycan 9, Glycan 6	**
PLEKHG2	T_{1195},S_{1197}	Glycan 3, Glycan 9	**
PLEKHG2	S_{14},S_{16}	Glycan 2, Glycan 7	**
LTBP2	T_{682},T_{684}	Glycan 8, Glycan 8	*****
NUTM1	S_{204},S_{207}	Glycan 7, Glycan 6	***
PODXL	S_{165},T_{167}	Glycan 9, Glycan 9	***
CASP8AP2	T_{1590}	Glycan 5	***
ANKHD1	S_{1266}	Glycan 9	**
CASP8AP2	S_{1868},T_{1872}	Glycan 9, Glycan 8	**
PSAP	T_{214},S_{216}	Glycan 7, Glycan 8	***
UBE2E1	T_{60},S_{68}	Glycan 9, Glycan 8	**
HECTD1	S_{707},S_{708}	Glycan 1, Glycan 5	***
C3	T_{77},T_{89}	Glycan 9, Glycan 2	***
GOLM1	T_{111},T_{112}	Glycan 9, Glycan 9	***
MUC19	T_{7512},T_{7513}	Glycan 9, Glycan 8	***
UBE2E1	T_{60},S_{68}	Glycan 8, Glycan 9	***
PSAP	S_{427},T_{428}	Glycan 8, Glycan 9	***
BASP1	S_{205}	Glycan 8	***
IRS2	T_{214},S_{222}	Glycan 8, Glycan 8	**
TFRC	T_{104},S_{106}	Glycan 8, Glycan 8	**
CARS2	S_{544},S_{545}	Glycan 8, Glycan 8	**
CCDC6	S_{352},S_{361}	Glycan 5, Glycan 2	**
IRS2	S_{237},T_{239}	Glycan 7, Glycan 2	****
NUCKS1	S_{214}	Glycan 8	****
PSMD3	S_{303}	Glycan 8	***
LAMB2	T_{1632},T_{1635}	Glycan 9, Glycan 6	**

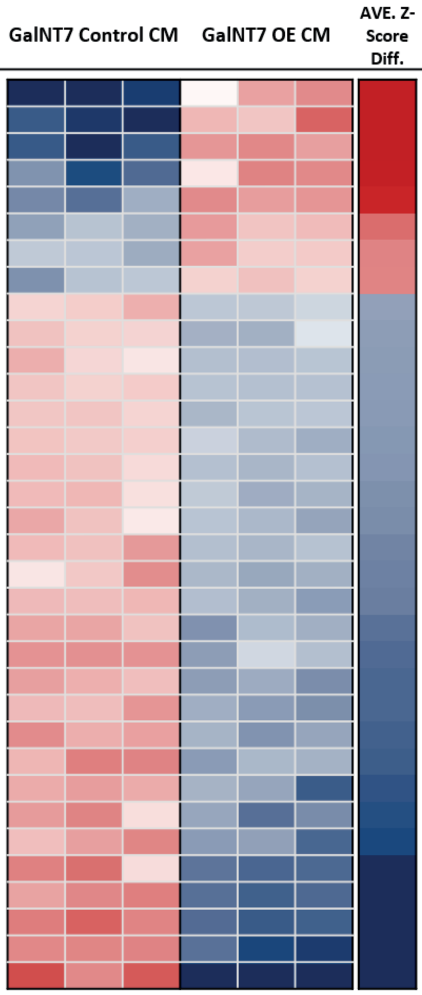

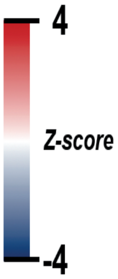

Glycan key:

Glycan Name	Compatible Structure
Glycan 1	
Glycan 2	
Glycan 3	
Glycan 4	
Glycan 5	
Glycan 6	
Glycan 7	
Glycan 8	
Glycan 9	

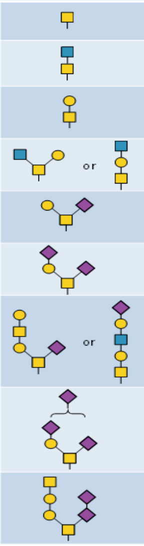

Supplement: Supplementary Figure 8 [file EMS162589-supplement-Supplementary_Figure_8.pdf]
