# Supplementary material for: Upregulation of GALNT7 in prostate cancer modifies O-glycosylation and promotes tumour growth
Source: Oncogene. Author manuscript; Available in PMC 2023 Mar 20. (PMC10020086; doi:10.1038/s41388-023-02604-x)

Supplementary Figure 9
GALNT7 regulates prostate cancer cell proliferation, migration and invasion in vitro

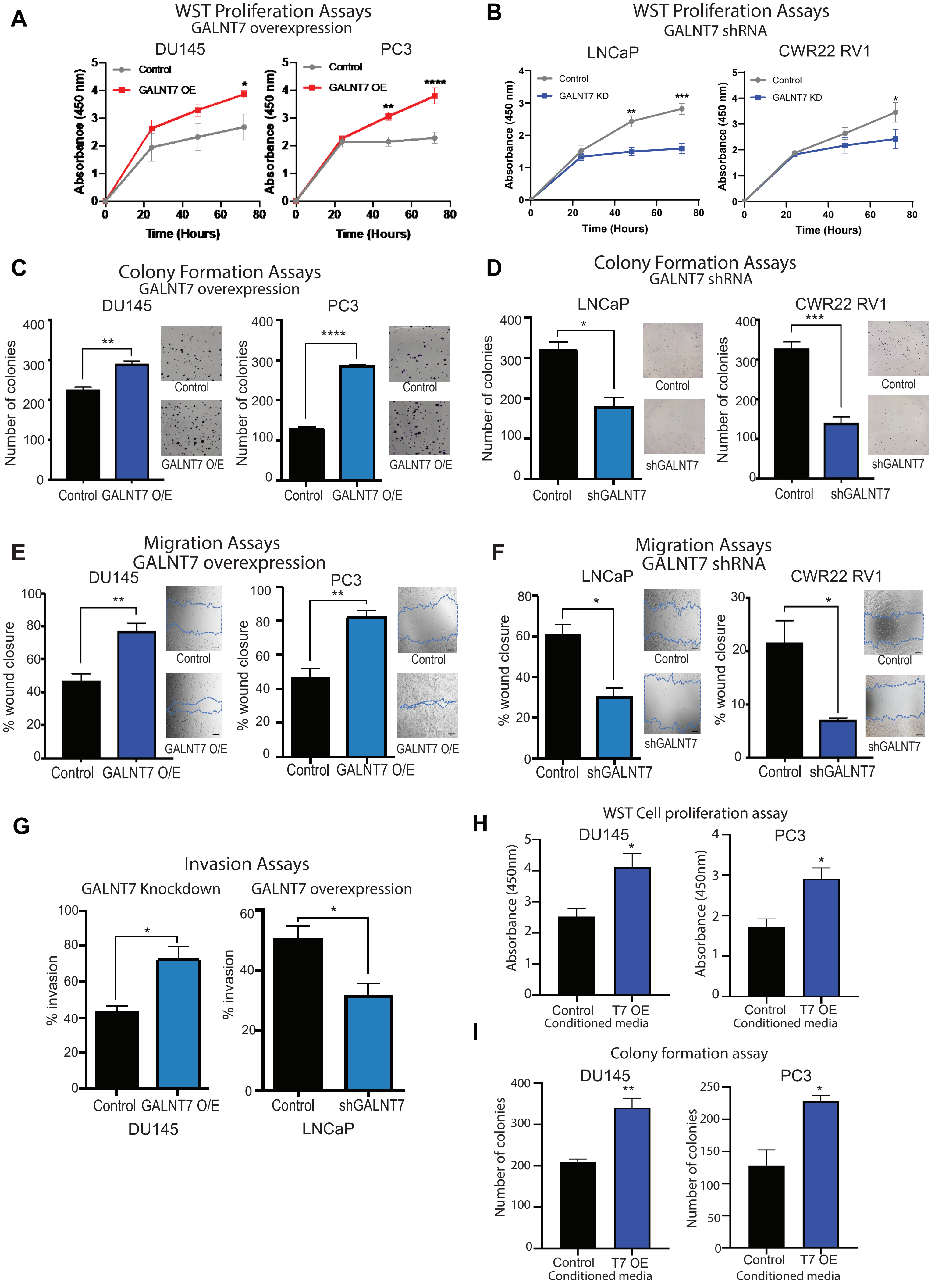

Supplement: Supplementary Figure 9 [file EMS162589-supplement-Supplementary_Figure_9.pdf]
