# Supplementary material for: Upregulation of GALNT7 in prostate cancer modifies O-glycosylation and promotes tumour growth
Source: Oncogene. Author manuscript; Available in PMC 2023 Mar 20. (PMC10020086; doi:10.1038/s41388-023-02604-x)
Supplement: Supplementary Figure legends [file EMS162589-supplement-Supplementary_Figure_legends.docx]

**Supplementary Figure legends**

**Supplementary Figure 1. GALNT7 is upregulated in prostate cancer tissue**. Immunohistochemistry analysis of GALNT7 protein in a 96 case TMA. GALNT7 levels are significantly higher in prostate cancer tissue relative to normal prostate tissue (n=96, unpaired t test, p=0.0124). Scale bar is 100µm.

**Supplementary Figure 2**. **Validation of GALNT7 antibody for immunohistochemistry.** (**A**) Blocking with GALNT7 immunising peptide inhibits GALNT7 antibody binding to prostate cancer tissue. (**B**) Immunohistochemistry staining of FFPE LNCaP cell pellets depleted of GALNT7 using siRNA. (**C**) Immunohistochemistry staining of FFPE DU145 cell pellets with upregulated GALNT7.

**Supplementary Figure 3**. **GALNT7 is detected in the blood and urine of men with prostate cancer.** (**A**) Validation of GALNT7 sandwich ELISA assays by detection of GALNT7 overexpression. (**B**) Serum PSA levels in 180 men taking part in the INNOVATE clinical trial ([31](#_ENREF_31)). (**C**) GALNT7 is also detected in urine samples from men with mCRPC (n=49).

**Supplementary Figure 4. *GALNT7* levels correlate with androgen receptor signalling in prostate cancer cells**. (**A,B**) Analysis of RNAseq data from the TCGA and SU2C patient cohorts ([24](#_ENREF_24), [33](#_ENREF_33)) shows a significant correlation between *GALNT7* and *AR* gene levels in both datasets. (**C**) Analysis of RNAseq data from 7 human prostate cancer patients pre- and post- androgen deprivation therapy (ADT) ([78](#_ENREF_78), [79](#_ENREF_79)) shows that there is a 2.6 fold downregulation of GALNT7 mRNA following ADT (p=0.00015, Mann Whitney U test). (**D**) RNAseq data from LTL331 patient-derived xenografts grown in mice ([80](#_ENREF_80)) show a reduction in GALNT7 *mRNA* levels following castration.

**Supplementary Figure 5. Validation of prostate cancer stable cell line models with knockdown or overexpression of GALNT7**. Real-time PCR (**A**) and western blot (**B**) analysis of GALNT7 mRNA and protein levels in four prostate cancer cell lines showed GALNT7 levels are higher in the AR+ cell lines LNCaP and CWR22RV1 compared to the AR- cell lines DU145 and PC3. (**C,D**) Knockdown of GALNT7 in LNCaP and CWR22Rv1 cells using shRNA mediated protein depletion was confirmed by real-time PCR and western blotting. (**E,F**) Overexpression of GALNT7 in DU145 and PC3 cells was also confirmed by real-time PCR and western blotting.

**Supplementary Figure 6. Lectin array and lectin flow cytometry analysis of prostate cancer cells.** (**A**) Lectin array profiling of PC3 prostate cancer cells indicates upregulation of GALNT7 increases binding of SBA lectin (which recognises Tn antigen), RCA-1 lectin (which recognises terminal galactose) and AAL lectin (which recognises fucose). (**B**) Increased binding of SBA lectin to cells with upregulated GALNT7 was confirmed by lectin flow cytometry.

**Supplementary Figure 7. Lectin and antibody profiling of prostate cancer extracellular vesicles (EVs)**. (**A**) Assay concept for the detection of EVs in conditioned media. (**B**) Comparison of EVs from cells lines identified 17 changes in lectin/antibody binding affected by GALNT7 KD or OE. (**C**) Analysis of the Tn expression of EVs from CWR22RV1 and PC3 cells confirms the effect of GALNT7 and the cancer-associated Tn antigen in prostate cancer.

**Supplementary Figure 8. Glycoproteomics of GALNT7 overexpressing prostate cancer cells.** (**A**) Glycoproteomics to map GALNT7 glycosylation sites on specific proteins in DU145 prostate cancer cells with upregulated GALNT7 identified 34 glycopeptides as specific substrates of GALNT7.

**Supplementary Figure 9. GALNT7 promotes prostate cancer cell proliferation, migration and invasion *in vitro***. (**A**) WST-1 cell proliferation assays show overexpression of GALNT7 in DU145 and PC3 cells significantly increases cell proliferation. (**B**) Conversely, knockdown of GALNT7 in LNCaP and CWR22RV1 cells significantly reduces cell proliferation. Overexpression and knockdown of GALNT7 also alters prostate cancer cell colony formation (**C,D**), migration (**E,F**) and invasion (**G**). (**H**,**I**) Conditioned media from DU145 and PC3 prostate cancer cells with upregulated GALNT7 significantly enhanced cell proliferation and colony formation of wildtype prostate cancer cells.

**Supplementary Figure 10. RNAseq analysis of prostate cancer cells with knockdown or overexpression of GALNT7.** (**A**) RNAseq identified 457 genes dynamically regulated by GALNT7 in prostate cancer cells. (**B**) Volcano plots of genes differentially expressed upon GALNT7 knockdown in CWR22RV1 cells and GALNT7 overexpression in DU145 cells. (**C**) Heatmap to show cell cycle genes that are reciprocally regulated in both RNAseq datasets. (**D**) GALNT7 overexpression reduces level of the tumour suppressor FOXO1 protein in prostate cancer. (**E**) Analysis of the TCGA PRAD cohort ([24](#_ENREF_24)) shows a significant correlation between *GALNT7* expression and the tumour suppressor gene *FOXO1* (n=549).
